# Leveraging deep learning to understand health beliefs about the Human Papillomavirus Vaccine from social media

**DOI:** 10.1038/s41746-019-0102-4

**Published:** 2019-04-15

**Authors:** Jingcheng Du, Rachel M. Cunningham, Yang Xiang, Fang Li, Yuxi Jia, Julie A. Boom, Sahiti Myneni, Jiang Bian, Chongliang Luo, Yong Chen, Cui Tao

**Affiliations:** 1 School of Biomedical Informatics, The University of Texas Health Science Center at Houston, Houston, TX USA; 2 Texas Children’s Hospital, Houston, TX USA; 3 School of Public Health, Jilin University, Changchun, China; 4 Baylor College of Medicine, Houston, TX USA; 5 Health Outcomes and Biomedical Informatics, College of Medicine, University of Florida, Gainesville, FL USA; 6 Perelman School of Medicine, The University of Pennsylvania, Philadelphia, PA USA; 7 Institute for Biomedical Informatics, The University of Pennsylvania, Philadelphia, PA USA; 8 Center for Evidence-based Practice, The University of Pennsylvania, Philadelphia, PA USA

**Keywords:** Health care, Cancer prevention

## Abstract

Our aim was to characterize health beliefs about the human papillomavirus (HPV) vaccine in a large set of Twitter posts (tweets). We collected a Twitter data set related to the HPV vaccine from 1 January 2014, to 31 December 2017. We proposed a deep-learning-based framework to mine health beliefs on the HPV vaccine from Twitter. Deep learning achieved high performance in terms of sensitivity, specificity, and accuracy. A retrospective analysis of health beliefs found that HPV vaccine beliefs may be evolving on Twitter.

## Introduction

The human papillomavirus (HPV) is the most common sexually transmitted disease and causes several types of cancers, including cervical, vaginal, vulvar, penile, anal, and oropharyngeal. Although the HPV vaccine is highly effective, vaccine refusal is common among parents of adolescents.^1^ Understanding parental beliefs about the HPV vaccine is an important step toward developing effective and targeted vaccine promotion strategies.^1,2^ The Health Belief Model (HBM) is the most widely used conceptual framework in health behavior research to explain why people adopt behaviors that lead to healthy lives.^3^ Studies have found that HBM constructs are associated with HPV vaccine intention and uptake.^4–6^

Traditional survey methods present significant limitations in assessing public health beliefs, including difficulties in reaching a large-scale population and tracking changes in real time.^7–9^ Social media enables millions of people to voluntarily and continuously share self-generated content, which allows access to the health beliefs of a large-scale population. Understanding the large amount of free text data on social media, however, requires advanced algorithms. Previous efforts were focused on developing traditional machine learning-based approaches to understand attitudes and health beliefs toward the HPV vaccine.^10–12^ Deep learning is a set of advanced computational models that has achieved state-of-the-art performance for various tasks in natural language understanding.^13–16^ The efficacy of deep-learning-based approaches to mining health beliefs about the HPV vaccine from Twitter discussions is unknown.

## Results

We focus on four primary HBM constructs: perceived susceptibility, perceived severity, perceived benefits, and perceived barriers. The inter-annotator agreements for the four HBM constructs are 0.727, 0.807, 0.831, and 0.834, respectively. Our deep-learning models achieved satisfactory results in terms of sensitivity, specificity, and accuracy on testing sets. The models achieved a mean accuracy of 80.50% for identifying HBM-related tweets and between 80.33% and 89.82% for the four HBM constructs. Table 1 shows the constructs, definition, sample tweets, and performance (estimated sensitivity, specificity, and accuracy, with their 95% confidence intervals) of the proposed deep-learning model.

**Table 1:** The annotation of HPV vaccination discussion on Twitter with respect to the four Health Belief Model (HBM) primary constructs and the performance of the deep-learning classifier on each annotation

Construct	Definition	Sample tweet	Sensitivity		Specificity		Accuracy	
			Mean	95% CI	Mean	95% CI	Mean	95% CI
Perceived susceptibility	The assessment of the risk of getting an HPV infection	hpv is so common almost everyone will be infected with the virus. but it can cause cancer. so why wait? vaccinate!	0.7418	0.7205–0.7630	0.9209	0.9131–0.9288	0.8937	0.8879–0.8996
Perceived severity	The assessment of whether an HPV infection is a sufficient health concern	learn about the human papillomavirus (hpv), which causes almost all cases of cervical cancer…	0.7561	0.7345–0.7776	0.9302	0.9238–0.9366	0.8958	0.8913–0.9002
Perceived benefits	The benefits of the HPV vaccine in protecting against HPV infection and, e.g., HPV infection-induced cancers	health lifestyle | here’s how the hpv vaccine can help cut the risk of cancer in gay men | news & gt	0.7289	0.7041–0.7536	0.9026	0.8952–0.9100	0.8589	0.8530–0.8648
Perceived barriers	The side effects of the HPV vaccines; cost of the vaccine; negative news reports on the vaccine	hpv vaccine is associated with serious health risks	0.8865	0.8763–0.8967	0.9086	0.9010–0.9163	0.8982	0.8944–0.9019
HBM related	Can be mapped to at least one of the above constructs	rate of teen boys being vaccinated against cancer-causing hpv is up	0.8050	0.7942–0.8157	0.8013	0.7884–0.8143	0.8033	0.7995–0.8071

*Definition* the explanation for HBM on the HPV vaccine, *CI* confidence interval

**Fig. 1:**
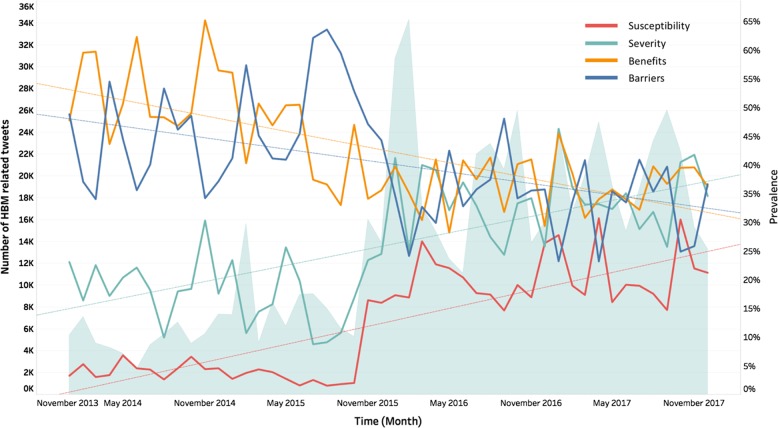
Retrospective analysis of health beliefs about the HPV vaccination, measured in each month. The shadowed area represents the total number of HBM-related tweets for each month, and the colored lines represent the prevalence of each HBM construct (defined by the ratio of the specific construct-related tweets to total HBM-related tweets)

After applying the model to classify the 956,262 un-labeled tweets, we classified 652,252 tweets, obtained from 216,864 unique Twitter user IDs, as HBM related. Among the related tweets, 184,604, 243,206, 373,228, and 309,501 tweets were categorized into the four primary HBM constructs, respectively. For each month from 2014 to 2017, we calculated the number of HBM-related tweets; we further defined the prevalence of each HBM construct by calculating the ratio of the number of tweets related to that construct to the total number of HBM-related tweets. Temporal analysis of the overall data (Fig. 1) showed that the prevalence of tweets in the *perceived susceptibility*/*severity* constructs increased every year, while tweets categorized into *perceived benefits*/*barriers* decreased.

A significant shift in health beliefs was seen in 2016. We checked the Twitter discussion as well as historical news media from 2016 and found that the significant shift was due largely to promotional articles on the HPV vaccine from several influential media sources, including the New York Times (“HPV Sharply Reduced in Teenage Girls Following Vaccine, Study Says,” 23 February 2016) and Time (“The HPV Vaccine Is Lowering Infection Rates,” 22 February 2016) as well as others. These articles led to a large proportion of the discussion at that time.

As can be seen in Fig. 1, two spikes in barriers were found in February and July in 2015. We reviewed the Twitter discussion during these two time periods and identified corresponding events that contributed to the high prevalence of barriers: The spike in February was due mainly to the Toronto Star’s story on Gardasil, titled, “A Wonder Drug’s Dark Side” (February 5, 2015), whereas the spike in that July was due mainly to the news that the European Medicines Agency was conducting a review of the HPV vaccine’s side effects.

## Discussion

We performed a retrospective analysis of HPV vaccine health beliefs, using Twitter data pulled from a large population. Our findings indicate that the number of tweets that correspond to certain HBM-related constructs have undergone a substantial temporal shift, which may indicate the evolving of HPV vaccine beliefs on Twitter. The decrease in the number of tweets related to perceived susceptibility/severity may reflect an improved understanding of the prevalence of HPV and HPV-related cancers as well as an increased awareness of the severity of these cancers. Likewise, the decrease in tweets related to perceived barriers may reflect a shift in parental assessment of the risk/benefit ratio in accepting the HPV vaccine for their teen. Specific events that may contribute to the changes in health beliefs were identified. Further analysis of the impact of these events could benefit the promotion of HPV vaccination. There are, however, certain limitations of our study. For example, our study did not consider information about the users and classified tweets independently. In the future, we plan to develop novel computational algorithms to understand health beliefs on the user level by analyzing the historical tweets for each user.

This study demonstrates the potential for utilizing social media to better understand HPV vaccine health beliefs. With deep-learning approaches, our study was able to map large-scale Twitter discussions on HPV vaccines to HBM constructs in a high accurate manner. Such deep-learning approaches can complement traditional surveys with real-time surveillance on the Twitter population.

## Methods

### Data collection and annotation

A combination of HPV vaccine-related keywords (i.e., HPV, human papillomavirus, Gardasil, and Cervarix) was used to collect 956,262 English-language tweets from 1 January 2014, to 31 December 2017, using Twitter streaming API (~1% of the entire stream volume). Three reviewers categorized a subset of 6000 tweets based on their relevance to the HBM constructs. Each tweet was assigned to none (not related to HBM), one, or multiple HBM constructs. The reviewers first annotated the same 500 tweets and resolved disagreements by discussion. Then, the reviewers categorized the remaining 5500 tweets independently. This manually categorized data set served as the gold-standard data for training and evaluation of the deep-learning model.

### Deep-learning model

**Fig. 2:**
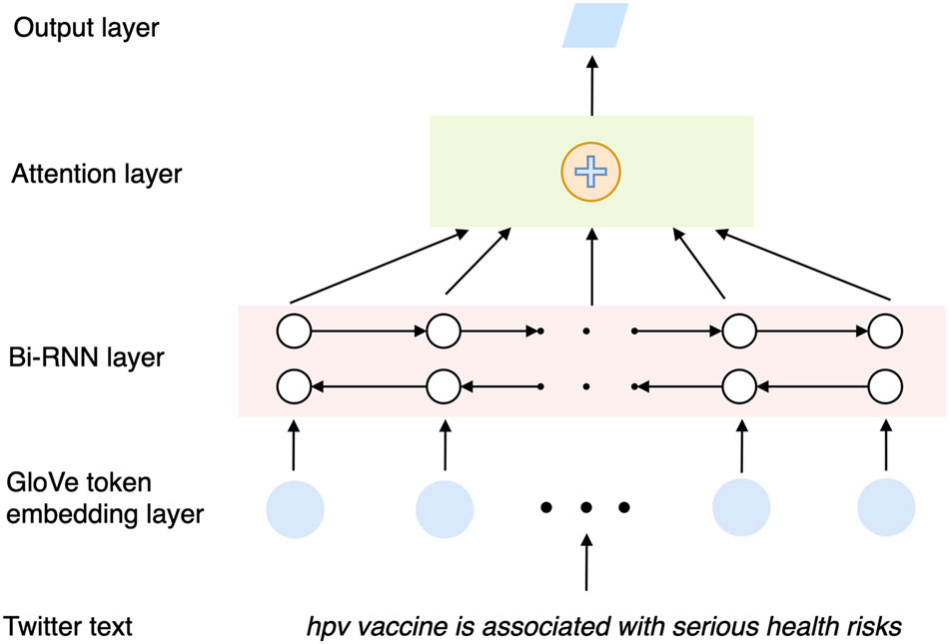
The architecture of the attentive recurrent neural network (RNN) for Twitter text classification

We frame the automatic categorization of tweets to the HBM constructs to text classification tasks. We propose an attentive recurrent neural network (RNN)-based deep-learning model for these tasks. The architecture of the proposed model can be seen in Fig. 2. Our model consists of four computation layers: (1) a token-embedding layer that maps each token (i.e., word) in the text to a 200-dimension vector; pre-trained Global Vectors for Word Representation (GloVe) Twitter (trained on 2 billion tweets)^17^ is used to initialize the token-embedding layer; (2) a bidirectional RNN (Bi-RNN) layer^18^ that takes the output of the token-embedding layer as the input and outputs a high-dimensional vector (length: 50) that represents the tweet content by capturing both forward and backward information from the text; (3) an attention layer^19^ that augments the bidirectional RNN layer by capturing salient information from the RNN output; and (4) a Softmax layer that normalizes the attention output into a probability distribution for classification.

We split the task into two steps: (1) categorize the tweet based on whether it is relevant to any of the HBM constructs (one classification task) and (2) categorize the relevant tweets into the four primary HBM constructs (four independent classification tasks). For Step 1, we divided all gold-standard tweets (6000 in total) into training, validation, and testing sets with a proportion of 7:1:2. For Step 2, we divided all HBM-related tweets (3264 in total) in the gold standard into training, validation, and testing sets with the same proportion. We performed hyper-parameter tuning on the validation set and evaluated the models on the testing sets. We repeated random sampling of the tweets 30 times with same proportion and calculated the sensitivity, specificity, and accuracy for each model at each time. We further calculated the mean and confidence interval of these values for each model. After the evaluation, we then applied one set of trained models to categorize the remaining un-labeled tweets into the four primary HBM constructs.

### Ethics approval and consent to participate

This study received expedited review and IRB approval from the Committee for the Protection of Human Subjects at The University of Texas Health Science Center at Houston. Waiver of informed consent was granted by the IRB due to the retrospective design of the study. The approved IRB protocol number is HSC-SBMI-16–0291.

## Data Availability

The data that support the findings of this study are available from the corresponding author upon request. The data are not publicly available due to privacy concerns for Twitter users.

## Code availability

The codes that support the findings of this study are available from the corresponding author upon request.
